# Synapse Density and Dendritic Complexity Are Reduced in the Prefrontal Cortex following Seven Days of Forced Abstinence from Cocaine Self-Administration

**DOI:** 10.1371/journal.pone.0102524

**Published:** 2014-07-29

**Authors:** Khampaseuth Rasakham, Heath D. Schmidt, Kevin Kay, Megan N. Huizenga, Narghes Calcagno, R. Christopher Pierce, Tara L. Spires-Jones, Ghazaleh Sadri-Vakili

**Affiliations:** 1 MassGeneral Institute for Neurodegenerative Disease, Massachusetts General Hospital, Boston, Massachusetts, United States of America; 2 Center for Neurobiology and Behavior, Department of Psychiatry, Perelman School of Medicine, University of Pennsylvania, Philadelphia, Pennsylvania, United States of America; University of Colorado, United States of America

## Abstract

Chronic cocaine exposure in both human addicts and in rodent models of addiction reduces prefrontal cortical activity, which subsequently dysregulates reward processing and higher order executive function. The net effect of this impaired gating of behavior is enhanced vulnerability to relapse. Previously we have shown that cocaine-induced increases in brain-derived neurotrophic factor (BDNF) expression in the medial prefrontal cortex (PFC) is a neuroadaptive mechanism that blunts the reinforcing efficacy of cocaine. As BDNF is known to affect neuronal survival and synaptic plasticity, we tested the hypothesis that abstinence from cocaine self-administration would lead to alterations in neuronal morphology and synaptic density in the PFC. Using a novel technique, array tomography and Golgi staining, morphological changes in the rat PFC were analyzed following 14 days of cocaine self-administration and 7 days of forced abstinence. Our results indicate that overall dendritic branching and total synaptic density are significantly reduced in the rat PFC. In contrast, the density of thin dendritic spines are significantly increased on layer V pyramidal neurons of the PFC. These findings indicate that dynamic structural changes occur during cocaine abstinence that may contribute to the observed hypo-activity of the PFC in cocaine-addicted individuals.

## Introduction

Alterations in structural plasticity within the reward circuitry are proposed to be key mechanisms contributing to cocaine's powerful ability to maintain drug-seeking behavior (reviewed in [1]). Previous studies have shown an increase in dendritic arborization and spine density in the nucleus accumbens (NAc) [2]–[4], ventral tegmental area [5], and the prefrontal cortex (PFC) [6] following exposure to cocaine. While most studies have focused on structural changes associated with the dysfunctional activity of the NAc, considerably fewer studies have examined the alterations in the PFC. Several lines of evidence demonstrate dysfunction of the PFC following chronic cocaine exposure in both human addicts [7], [8] and in rodent models of addiction [9], [10]. Therefore, characterizing the structural changes that occur in the PFC is pertinent to understanding the molecular events that underlie addiction.

The PFC regulates impulse control and decision-making and thus plays an important role in an individual's ability to control behavior, particularly in drug dependence [8], [11]. For example, in individuals addicted to cocaine, decreased prefrontal cortex activation is associated with drug withdrawal and disrupted higher order executive responses [7], [8], which can enhance vulnerability to relapse. In rodents, increased neuronal activity in the PFC is associated with cocaine intake [9], [10], compulsive drug-seeking behavior [12], and cocaine reinstatement after withdrawal [13]–[15]. In addition, membrane bistability is abolished in the PFC following chronic cocaine administration [16]. Finally, drug-induced metabolic activity in the PFC is blunted in rats administered a challenge injection during withdrawal from cocaine self-administration [9], [17]. Together, these studies indicate that chronic cocaine induces profound functional changes in PFC that may be associated with an increase in the number of inhibitory synapses and/or a reduction in excitatory synapses in the PFC. However, the morphological alterations that occur in the PFC following chronic drug use have not been elucidated.

In the present study we sought to examine whether abstinence from cocaine leads to structural changes in the PFC. Morphological alterations were examined using a traditional method, Golgi staining, as well as a novel technique, array tomography. Array tomography is a unique method that combines ultrathin tissue sectioning with immunofluorescence and three-dimensional image reconstruction to allow accurate quantification of total and sub-type specific synapse density [18], [19]. Using these methods, our results indicated substantial plasticity in rat PFC in response to abstinence form cocaine.

## Materials and Methods

### Animals and housing

Male Sprague-Dawley rats (Rattus norvegicus) weighing 250–300 g were obtained from Taconic Laboratories (Germantown, NY). Animals were individually housed with food and water available ad libitum in their home cage. The experimental protocols were all consistent with the guidelines issued by the U.S. National Institutes of Health and were approved by the Perelman School of Medicine at the University of Pennsylvania and the University of Pennsylvania's Institutional Animal Care and Use Committee.

### Surgery

Prior to surgery, the rats were anesthetized with 80 mg/kg ketamine and 12 mg/kg xylazine (i.p.; Sigma-Aldrich, St. Louis, MO). An indwelling silastic catheter (inner diameter 0.33 mm, outer diameter 0.64 mm) was inserted into the right jugular vein and sutured in place. The catheter was then passed subcutaneously over the shoulder blade and routed to a mesh backmount platform (CamCath, Cambridge, UK) that was sutured below the skin directly above the scapulae. Catheters were flushed daily with 0.3 ml of the antibiotic Timentin (ticarcillin disodium/potassium clavulanate, 0.93 mg/ml; Henry Schein, Melville, NY) dissolved in heparinized saline (10 U/ml). The catheters were sealed with plastic obturators when not in use.

### Cocaine self-administration

Rats were allowed 7 days to recover from surgery before cocaine self-administration commenced. Rats were randomly assigned to one of two groups: cocaine self-administering animals and yoked saline controls. Each rat trained to respond for contingent cocaine infusions was paired with a yoked subject that received the same number and temporal pattern of infusions as self-administered by the paired cocaine-experimental rat. Lever pressing for the saline-yoked rats had no scheduled consequences.

Initially, cocaine-experimental rats were placed in the modular operant chambers (Med Associates, St. Albans, VT) and allowed to lever press for intravenous cocaine infusions (0.25 mg cocaine/59 µl saline, infusion over 5 s) on a fixed-ratio 1 (FR1) schedule of reinforcement. Once a cocaine-experimental rat achieved at least 20 infusions of cocaine in a single operant session under the FR1 schedule, the response requirement was switched to a FR5 schedule of reinforcement. For responding on both fixed ratio schedules, the maximum number of cocaine infusions was limited to 30 per daily self-administration session and a 20 s time-out period followed each cocaine infusion, during which time active lever responses were tabulated but had no scheduled consequences. Daily 2 h operant sessions (7 days/week) were conducted for a total of 14 days. Responses made on the inactive lever, which had no scheduled consequences, were also recorded during both the FR1 and FR5 training sessions.

After the 14^th^ daily operant session, cocaine-experimental and yoked saline control rats were returned to their home cages where they underwent 7 days of forced drug abstinence. On the 7^th^ day of cocaine abstinence, brains were removed and the PFC was dissected on ice. Seven days of cocaine abstinence were chosen in order to draw direct comparisons to our previously published study examining cocaine-induced changes in PFC BDNF expression [20].

### Perfusion

Rats were anesthesized (100 mg/kg, i.p. sodium pentobarbital) and perfused with ice-cold 4% paraformaldehyde in 0.1 M PB, pH 7.4 (PFA). One hemisphere from each brain was used for golgi staining and the other hemisphere for array tomography. Array hemispheres were post-fixed in 4% PFA with 2.5% sucrose for 2 hours and Golgi hemispheres were post fixed for 48 h in 4% PFA.

### Array Tomography

Array tomography experiments were performed as previously described [19], [21]. Briefly, PFA fixed tissue was embedded in resin and coronal (70 nm) sections at the level of the mPFC were cut and collected as a ribbon. Ribbons were hydrated in 50 mM glycine in Tris and blocked in blocking solution (0.05% Tween/0.1% bovine serum albumin in Tris buffer (50 mM Tris/150 mM NaCl, pH 7.6). The ribbons were stained with primary antibodies, GAD65 (Chemicon), PSD95 (Cell Signaling), or synaptophysin (Abcam), in blocking solution overnight at 4°C. Ribbons were washed with Tris buffer and stained with secondary antibodies at 1∶50 in blocking solution (goat anti-mouse Alexa-flour 488 and goat anti-rabbit cy3 or donkey anti-rabbit cy5). Ribbons were counter stained with DAPI to facilitate finding the same sites on every section. Tile-scan images were collected using a Zeiss AxioImager Z2 epifluorescence microscope. Images from the same site on each of 20–30 serial sections per ribbon were acquired at 63x with automated programs specialized for array tomography.

### Array Tomography Analysis

The serial images from each ribbon were opened sequentially, converted to a stack and aligned with the plugins MultiStackReg and StackReg (courtesy of B. Busse at Stanford University and [21], [22]. Crop boxes (19.5 µmx19.5 µm) were used to select regions of interest (ROI) in the neuropil for quantification. Selections had to exclude neuronal cell bodies or other obscuring features. For automated image analysis, crops of interest (or ROIs) for synaptophysin, glutamic acid decarboxylase-65 (GAD65) and PSD95 were automatically thresholded respectively with automated algorithms in ImageJ. Crops were coded and analysis was run blind to condition. An automated, threshold-based detection program to quantify the number of puncta identified as positive synapses was used as previously described [23]. Densities of presynaptic terminals, excitatory postsynaptic terminals, and the percentage of GAD-positive (inhibitory) synapses were calculated from an average of 75 sample sites per animal collected from two different tissue blocks from the PFC (n = 5 cocaine treated, 5 saline treated animals) for a total of 29,154 postsynaptic puncta and 53,565 presynaptic puncta from 818 sampling sites across the 5 saline treated animals and 29,662 postsynaptic and 17,034 presynaptic puncta from 588 sampling sites across the 5 cocaine treated animals. The median values for synapse density and the percentage of inhibitory synapses per animal were calculated and t-tests run using the animal medians to test whether there was a difference between group means.

### Rapid-Golgi Method

Single section Golgi staining was performed as described previously [24], [25]. Briefly, the mPFC from one hemisphere of each animal was cut into 100 µm coronal sections and post-fixed in 1% osmium tetraoxide followed by three washes in 0.1 M PB, pH 7.4. Sections were incubated in 3.5% potassium dichromate overnight, washed briefly and infiltrated with 1.5% silver nitrate by the sandwich method [25]. Sections were mounted on gelatin-coated slides with 20% sucrose and dehydrated through a series of alcohol concentrations followed by de-fatting in xylene and coverslipping.

### Golgi Analysis

Golgi slides were coded and analyzed blind to condition and all analyzed by the same experimenter. Neuronal images and tracings and representative images of dendritic spines were collected using an upright BX51 Olympus microscope with an integrated motorized stage (Prior Scientific, Rockland, MA) with a 20×0.7 NA objective. For dendritic branching analysis, 7 neurons were selected for analysis per animal. We measured neurite length and complexity using the macros NeuronJ and Advanced Sholl Analysis, respectively. The number of intersections (branch points) within concentric circles at radii between 5–250 µm (including basal and apical dendrites) were measured and compared between groups. For spine density analysis, 4–5 segments of at least 20 µm in length from third order basal dendrites were analyzed per neuron from 5–7 neurons per animal using a Zeiss AxioImager Z2 epifluorescence microscope with a 63x oil-immersion objective. Spine morphology was classified as described previously [26]. Linear spine density for each dendritic segment and spine morphology (thin, stubby, mushroom, cup-shaped) of each spine were compared between groups. Open source software from National Institutes of Health (ImageJ) was used for Golgi and array tomography data analysis.

## Results

### Abstinence from cocaine reduces total synapse density

**Figure 1:**
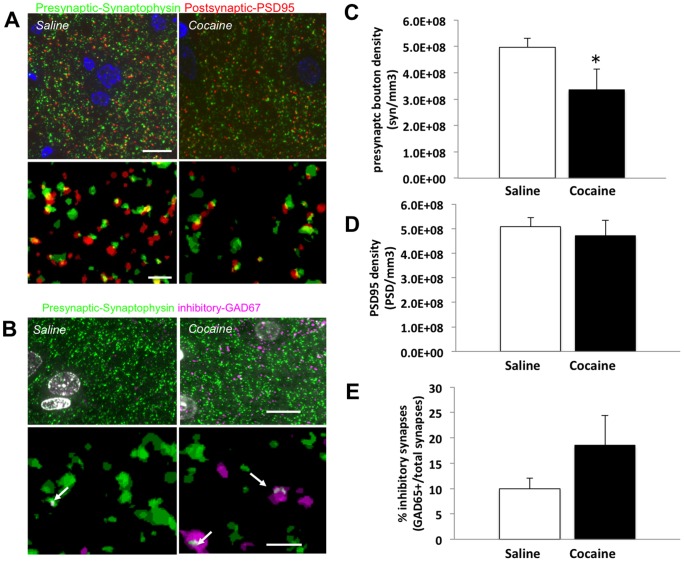
Array tomography reveals alterations in synapse density in the PFC following 7 days of abstinence from cocaine. (**A**) Ribbons of 70 nm sections were stained with postsynaptic marker PSD95 (red), presynaptic marker synaptophysin (green), and DAPI to label nuclei (blue) (**B**) Another set of ribbons were stained with synaptophysin (green) and GAD65 (magenta) to determine the percentage of inhibitory synapses. Raw images of single sections from saline (left) and cocaine treated (right) rats are shown in the top row of panels **A** and **B**. Images were aligned, processed to remove noise, and synapses detected in three-dimensional volumes as shown in the 3D rendering of 5 serial sections (bottom rows **A** and **B**) (**C**) Quantification of presynaptic terminals (**D**) Quantification of PSD95 positive postsynaptic terminals (**E**) Quantification of GAD65 positive terminals. Graphs show mean + S.E.M. * p<0.05 t-test; n = 5 animals/group; scale bar  = 10 µm top panels, and 2 µm bottom panels.

**Figure 2:**
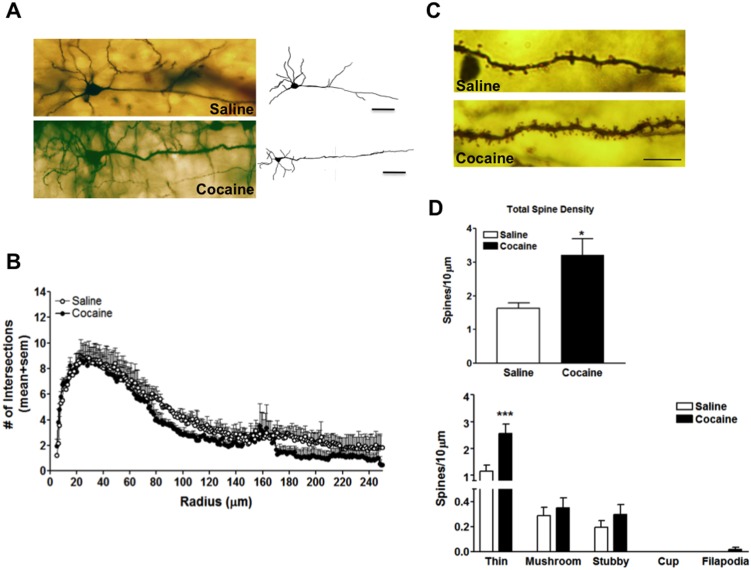
Single section Golgi analysis reveals alterations in dendritic branching and spine formation in the PFC following 7 days of abstinence from cocaine. (**A**) Representative photomicrograph of a Golgi stain pyramidal neuron and tracing from yoked-saline control (top left, top right) and cocaine (bottom left, bottom right) treated rats scale bar  = 50 µm (**B**) Sholl plot analysis of animals self-administering saline (open circles) and cocaine (black-filled circles) (**C**) Representative basal dendritic segment from a yoked-saline (top) and cocaine (bottom) neuron scale bar  = 10 µm (**D**) Quantitative analysis of total spine number and spine density classified by spine sub-type from 4–5 segments from 5–7 neurons from each animal, n = 4 animals/group. *, *p*<0.05; ***, *p*<0.001.

Array tomography was used to measure changes in both excitatory and inhibitory synapses in order to determine the specific morphological alterations that occur in the PFC in response to abstinence from cocaine self-administration. Array tomography is a high-throughput method that allows for the accurate quantification of total, inhibitory, and excitatory synapses in structures that are too small to be properly identified or localized with traditional confocal microscopy methods [19]. As both inhibitory and excitatory synapses are essential components of the drug reward circuitry [13], [27], [28] we used this novel methodology to assess morphological changes in the PFC during abstinence from cocaine. Seventy nm PFC sections from one brain hemisphere of 5 yoked-saline and 5 cocaine-experienced rats were stained with antibodies to PSD95, a postsynaptic excitatory marker, synaptophysin, a presynaptic marker, and GAD65, which labeled inhibitory neurons and synapses. Synapse densities and the percentage of inhibitory synapses were determined in cortical layer V (Figure 1A and 1B). Our results indicate that during abstinence from cocaine there was a significant decrease in synaptophysin density (Figure 1C), which measures all presynaptic terminals [t(7) = 2, p<0.05]. There was no significant decrease in excitatory synapse density [t(8) = 0.48, p = 0.32] as measured by counting PSD95 puncta (Figure 1D). Interestingly, there was a non-significant trend toward an increase in the percentage of GAD65-positive inhibitory synapses [t(8) = −1.39, p = 0.9] (Figure 2E).

### Abstinence from cocaine reduces dendritic branching while transiently increasing spine density in the PFC

The Golgi method was used to examine alterations in neuronal branching and dendritic spine density in order to confirm the ultrastructural changes observed in synapse density (Figure 1). We performed single section rapid Golgi impregnation on a subset of neurons in the PFC from the other hemispheres of the same animals that were used for the array tomography studies. Dendritic branching, dendritic spine counts, and spine morphology were assessed. Two representative pyramidal neurons from the PFC of a yoked-saline control and a cocaine-exposed rat are shown in Figure 2A. Sholl plot measured the number of intersections (branch points) within concentric circles at radii between 5–250 µm. Our results demonstrate that following 7 days of forced abstinence from cocaine self-administration there was a significant reduction in dendritic complexity (Figure 2B). Two-way repeated measures ANOVA analysis of sholl plot data revealed significant main effects of treatment [F_(1,738)_ = 30.59, p<0.0001] and radius [F_(245, 738)_ = 289.6, p<0.0001] (Figure 2B), confirming a loss of dendrites that agree with the loss of synapses measured in array studies (Figure 1C). Analysis of second and third order basilar dendrites revealed a significant increase in dendritic spines following 7 days of cocaine abstinence [t(6) = −3.12, p<0.05] (Figure 2D). More specifically, abstinence from cocaine exposure increased the number of thin spine subtype, while having no significant effect on other spine subtypes (Figure 2E), as revealed by two way repeated measures ANOVA with main effects of treatment [F_(1,30)_ = 11.9, p = 0.0017], spine subtype [F_(4,30)_ = 57.7, p<0.0001], and a significant treatment x spine subtype interaction [F_(1, 4, 30)_ = 8.8, p<0.0001].

## Discussion

In the present study we demonstrate that there are pronounced structural and synaptic changes in layer V of the PFC following 7 days of forced abstinence from cocaine self-administration. Specifically, there is a significant decrease in dendritic branching of pyramidal neurons and a general loss in synapse density as measured by decreased density of overall presynaptic boutons labeled with synaptophysin. Despite the loss of presynaptic density, basal dendrites of layer V pyramidal neurons underwent an increase in dendritic spine density, particularly of thin, plastic spines. As we did not detect significant changes in density of PSD95, it can be speculated that decreases in presynaptic terminals but increase in spine density may be due to an increase in number of multi-synaptic boutons. Additionally, it is also worth noting that we observed a trend towards increased inhibitory synapses in the PFC. Since thin spines are involved in plasticity [29], the increase in these spines could represent compensatory plasticity to maintain synaptic inputs on these denervated neurons which have lost dendritic branches.

Previous studies demonstrated that cocaine increases dendritic arborization and spine density in the NAc [2]–[4]. Recently, Dumitriu et al., 2012 [30] demonstrated that cocaine dynamically alters proximal spines in the NAc core and shell. Specifically, in the shell, withdrawal from cocaine increased thin spines, while decreasing mushroom spine head density in the NAc shell [30]. In contrast to studies of the NAc, there are only a few studies that have examined cocaine's effects on neuronal morphology in the PFC [6], [31]. Our data are consistent with a recent study demonstrating that cocaine induces an increase in spine density in the PFC [31]. Notably, mice that had a greater increase in persistent and stable spines, i.e. spines present 3 days post-withdrawal, on apical dendrites showed higher cocaine conditioned place preference scores and cocaine-induced hyperactivity [31]. A previous study in rat PFC layer II-III neurons reported values of approximately 3 spines per µm of dendrite on both apical and basal dendrites, a surprisingly dense level of spines that was modifiable by stress [32]. Our values in control rats of ∼2 spines/10 µm of dendritic segments are lower, which may be due to the different neuronal population analyzed (layer V basal dendrites) or to the difference in imaging technique. In the present study we used the single section rapid Golgi staining method while iontophoretic injections of Lucifer yellow dye combined with confocal imaging were used by Radley and colleagues [32] to visualize neuronal and dendritic morphology. In addition, our findings also highlight the importance of the duration of abstinence from cocaine self-administration that lead to structural changes in the brain. A previously published report demonstrated an increase in dendritic arborization following long-term (24–25 days) withdrawal from cocaine in female rats [6], in contrast to our observed decrease following 7 days of forced abstinence in male rats. Despite these methodological differences and differences in branching data, increased number of spines was observed in both studies, confirming large-scale circuit reorganization during cocaine abstinence. Future studies will elucidate the time course of these events in order to determine whether these structural changes are transient or long-lasting.

Our findings indicate that forced abstinence from cocaine self-administration induces dynamic structural changes and causes synaptic re-organization in the PFC. These results may explain the hypo-activity in the PFC that occurs as a result of repeated cocaine exposure [8], [33]. Furthermore, our findings support previous studies demonstrating deactivation of the PFC [7], [8], and increased extracellular GABA in the medial PFC during cocaine withdrawal [34]. Thus, the mechanisms accounting for PFC hypo-activity following chronic cocaine exposure [8], [10] may include (1) an increase in GABAergic, (2) reduction in glutamatergic and/or (3) reduction in dopaminergic synaptic input to the PFC. The present study demonstrates that abstinence from cocaine significantly reduces overall synapse density as indicated by a reduction in the number of synaptophysin positive synaptic puncta. These data suggest that there is a reduction in post-synaptic responsiveness in the PFC, possibly mediated by decreased glutamate or dopamine input. Indeed, there are studies indicating that cocaine induces reductions in glutamatergic tone [35], [36]. However, using the Golgi method, we observed an increase in the number of thin dendritic spines on basal dendrites of pyramidal neurons, which suggests an increase in excitatory input in the PFC to remaining neurites. These apparently conflicting data may reflect an overall loss of synapses associated with the large loss of dendrites we observe with a compensatory response, possibly mediated by increased BDNF, as shown in our previous findings [20], to increase the density of dendritic spines on remaining neurites.

Collectively, our findings indicate dynamic re-organization in the PFC during cocaine abstinence. Specifically, there is a significant reduction in synaptic connectivity, loss of dendritic branching and an increase in the number of thin spines in the rat PFC following 7 days of forced drug abstinence from cocaine self-administration. These results may provide the structural basis for the observed hypo-activity observed in the PFC of chronic cocaine abusers and perhaps explain the loss of cognitive control that occurs during cocaine addiction.

## References

[1] Dietz DM, Dietz KC, Nestler EJ, Russo SJ (2009) Molecular mechanisms of psychostimulant-induced structural plasticity. Pharmacopsychiatry 42 Suppl 1S69–78. 19434558 10.1055/s-0029-1202847 PMC2734446

[2] Lee KW, Kim Y, Kim AM, Helmin K, Nairn AC, et al (2006) Cocaine-induced dendritic spine formation in D1 and D2 dopamine receptor-containing medium spiny neurons in nucleus accumbens. Proc Natl Acad Sci U S A 103: 3399–3404. 16492766 10.1073/pnas.0511244103 PMC1413917

[3] Norrholm SD, Bibb JA, Nestler EJ, Ouimet CC, Taylor JR, et al (2003) Cocaine-induced proliferation of dendritic spines in nucleus accumbens is dependent on the activity of cyclin-dependent kinase-5. Neuroscience 116: 19–22. 12535933 10.1016/s0306-4522(02)00560-2 PMC4296576

[4] Robinson TE, Gorny G, Mitton E, Kolb B (2001) Cocaine self-administration alters the morphology of dendrites and dendritic spines in the nucleus accumbens and neocortex. Synapse 39: 257–266. 11169774 10.1002/1098-2396(20010301)39:3<257::AID-SYN1007>3.0.CO;2-1

[5] Sarti F, Borgland SL, Kharazia VN, Bonci A (2007) Acute cocaine exposure alters spine density and long-term potentiation in the ventral tegmental area. Eur J Neurosci 26: 749–756. 17686047 10.1111/j.1460-9568.2007.05689.x

[6] Robinson TE, Kolb B (1999) Alterations in the morphology of dendrites and dendritic spines in the nucleus accumbens and prefrontal cortex following repeated treatment with amphetamine or cocaine. Eur J Neurosci 11: 1598–1604. 10215912 10.1046/j.1460-9568.1999.00576.x

[7] Bolla K, Ernst M, Kiehl K, Mouratidis M, Eldreth D, et al (2004) Prefrontal cortical dysfunction in abstinent cocaine abusers. J Neuropsychiatry Clin Neurosci 16: 456–464. 15616172 10.1176/appi.neuropsych.16.4.456 PMC2771441

[8] Goldstein RZ, Volkow ND (2002) Drug addiction and its underlying neurobiological basis: neuroimaging evidence for the involvement of the frontal cortex. Am J Psychiatry 159: 1642–1652. 12359667 10.1176/appi.ajp.159.10.1642 PMC1201373

[9] Chen YI, Famous K, Xu H, Choi JK, Mandeville JB, et al (2011) Cocaine self-administration leads to alterations in temporal responses to cocaine challenge in limbic and motor circuitry. Eur J Neurosci 34: 800–815. 21896062 10.1111/j.1460-9568.2011.07806.x PMC3172610

[10] Sun W, Rebec GV (2006) Repeated cocaine self-administration alters processing of cocaine-related information in rat prefrontal cortex. J Neurosci 26: 8004–8008. 16870745 10.1523/JNEUROSCI.1413-06.2006 PMC6674208

[11] Volkow ND, Fowler JS (2000) Addiction, a disease of compulsion and drive: involvement of the orbitofrontal cortex. Cereb Cortex 10: 318–325. 10731226 10.1093/cercor/10.3.318

[12] Jentsch JD, Taylor JR (1999) Impulsivity resulting from frontostriatal dysfunction in drug abuse: implications for the control of behavior by reward-related stimuli. Psychopharmacology (Berl) 146: 373–390. 10550488 10.1007/pl00005483

[13] McFarland K, Kalivas PW (2001) The circuitry mediating cocaine-induced reinstatement of drug-seeking behavior. J Neurosci 21: 8655–8663. 11606653 10.1523/JNEUROSCI.21-21-08655.2001 PMC6762812

[14] McFarland K, Lapish CC, Kalivas PW (2003) Prefrontal glutamate release into the core of the nucleus accumbens mediates cocaine-induced reinstatement of drug-seeking behavior. J Neurosci 23: 3531–3537. 12716962 10.1523/JNEUROSCI.23-08-03531.2003 PMC6742291

[15] Winstanley CA, Green TA, Theobald DE, Renthal W, LaPlant Q, et al (2009) DeltaFosB induction in orbitofrontal cortex potentiates locomotor sensitization despite attenuating the cognitive dysfunction caused by cocaine. Pharmacol Biochem Behav 93: 278–284. 19135469 10.1016/j.pbb.2008.12.007 PMC2820241

[16] Trantham H, Szumlinski KK, McFarland K, Kalivas PW, Lavin A (2002) Repeated cocaine administration alters the electrophysiological properties of prefrontal cortical neurons. Neuroscience 113: 749–753. 12182882 10.1016/s0306-4522(02)00246-4 PMC5509069

[17] Lu H, Chefer S, Kurup PK, Guillem K, Vaupel DB, et al (2012) fMRI response in the medial prefrontal cortex predicts cocaine but not sucrose self-administration history. Neuroimage 62: 1857–1866. 22664568 10.1016/j.neuroimage.2012.05.076 PMC3875563

[18] Micheva KD, Busse B, Weiler NC, O'Rourke N, Smith SJ (2010) Single-synapse analysis of a diverse synapse population: proteomic imaging methods and markers. Neuron 68: 639–653. 21092855 10.1016/j.neuron.2010.09.024 PMC2995697

[19] Micheva KD, Smith SJ (2007) Array tomography: a new tool for imaging the molecular architecture and ultrastructure of neural circuits. Neuron 55: 25–36. 17610815 10.1016/j.neuron.2007.06.014 PMC2080672

[20] Sadri-Vakili G, Kumaresan V, Schmidt HD, Famous KR, Chawla P, et al (2010) Cocaine-induced chromatin remodeling increases brain-derived neurotrophic factor transcription in the rat medial prefrontal cortex, which alters the reinforcing efficacy of cocaine. J Neurosci 30: 11735–11744. 20810894 10.1523/JNEUROSCI.2328-10.2010 PMC2943400

[21] Koffie RM, Meyer-Luehmann M, Hashimoto T, Adams KW, Mielke ML, et al (2009) Oligomeric amyloid beta associates with postsynaptic densities and correlates with excitatory synapse loss near senile plaques. Proc Natl Acad Sci U S A 106: 4012–4017. 19228947 10.1073/pnas.0811698106 PMC2656196

[22] Thevenaz P, Ruttimann UE, Unser M (1998) A pyramid approach to subpixel registration based on intensity. IEEE Trans Image Process 7: 27–41. 18267377 10.1109/83.650848

[23] Kopeikina KJ, Carlson GA, Pitstick R, Ludvigson AE, Peters A, et al (2011) Tau accumulation causes mitochondrial distribution deficits in neurons in a mouse model of tauopathy and in human Alzheimer's disease brain. Am J Pathol 179: 2071–2082. 21854751 10.1016/j.ajpath.2011.07.004 PMC3181340

[24] Gabbott PL, Somogyi J (1984) The ‘single’ section Golgi-impregnation procedure: methodological description. J Neurosci Methods 11: 221–230. 6392756 10.1016/0165-0270(84)90084-0

[25] Izzo PN, Graybiel AM, Bolam JP (1987) Characterization of substance P- and [Met]enkephalin-immunoreactive neurons in the caudate nucleus of cat and ferret by a single section Golgi procedure. Neuroscience 20: 577–587. 2438593 10.1016/0306-4522(87)90111-4

[26] Spires TL, Grote HE, Garry S, Cordery PM, Van Dellen A, et al (2004) Dendritic spine pathology and deficits in experience-dependent dendritic plasticity in R6/1 Huntington's disease transgenic mice. European Journal of Neuroscience 19: 2799–2807. 15147313 10.1111/j.0953-816X.2004.03374.x

[27] Kalivas PW, O'Brien C (2008) Drug addiction as a pathology of staged neuroplasticity. Neuropsychopharmacology 33: 166–180. 17805308 10.1038/sj.npp.1301564

[28] Pierce RC, Reeder DC, Hicks J, Morgan ZR, Kalivas PW (1998) Ibotenic acid lesions of the dorsal prefrontal cortex disrupt the expression of behavioral sensitization to cocaine. Neuroscience 82: 1103–1114. 9466434 10.1016/s0306-4522(97)00366-7

[29] Bourne J, Harris KM (2007) Do thin spines learn to be mushroom spines that remember? Curr Opin Neurobiol 17: 381–386. 17498943 10.1016/j.conb.2007.04.009

[30] Dumitriu D, Laplant Q, Grossman YS, Dias C, Janssen WG, et al (2012) Subregional, dendritic compartment, and spine subtype specificity in cocaine regulation of dendritic spines in the nucleus accumbens. J Neurosci 32: 6957–6966. 22593064 10.1523/JNEUROSCI.5718-11.2012 PMC3360066

[31] Munoz-Cuevas FJ, Athilingam J, Piscopo D, Wilbrecht L (2013) Cocaine-induced structural plasticity in frontal cortex correlates with conditioned place preference. Nat Neurosci 16: 1367–1369. 23974707 10.1038/nn.3498 PMC3940437

[32] Radley JJ, Rocher AB, Miller M, Janssen WG, Liston C, et al (2006) Repeated stress induces dendritic spine loss in the rat medial prefrontal cortex. Cereb Cortex 16(3): 313–320. 15901656 10.1093/cercor/bhi104

[33] Volkow ND, Mullani N, Gould KL, Adler S, Krajewski K (1988) Cerebral blood flow in chronic cocaine users: a study with positron emission tomography. Br J Psychiatry 152: 641–648. 3262397 10.1192/bjp.152.5.641

[34] Jayaram P, Steketee JD (2005) Effects of cocaine-induced behavioural sensitization on GABA transmission within rat medial prefrontal cortex. Eur J Neurosci 21: 2035–2039. 15869498 10.1111/j.1460-9568.2005.04000.x

[35] Madayag A, Lobner D, Kau KS, Mantsch JR, Abdulhameed O, et al (2007) Repeated N-acetylcysteine administration alters plasticity-dependent effects of cocaine. J Neurosci 27: 13968–13976. 18094234 10.1523/JNEUROSCI.2808-07.2007 PMC2996827

[36] Miguens M, Del Olmo N, Higuera-Matas A, Torres I, Garcia-Lecumberri C, et al (2008) Glutamate and aspartate levels in the nucleus accumbens during cocaine self-administration and extinction: a time course microdialysis study. Psychopharmacology (Berl) 196: 303–313. 17940751 10.1007/s00213-007-0958-x

